# Filling Teeth

**Published:** 1864-04

**Authors:** W. H. Shadoan


### FILLING TEETH.

Read before the Mississipi Valley Dental Association, February 18, 1864.

BY W. H. SHADOAN.

MR. PRESIDENT AND GENTLEMEN.—The subject upon which I design occupying your attention for a short time is filling teeth with gold.

What I shall say on the subject may not interest this Association, yet it may reach some one that it will interest, and perchance do him good, if so, then I am content.

Filling decayed teeth is a department within itself in which is involved, a greater variety of operations than any other, and as each of these efforts involve in a great degree the comfort of the patient, as well as the professional reputation of the dentist, we conclude that anything that tends to improve the manner, style and durability of his operations, will be of interest to him.

It is not my desire to enter into the minutiæ of all "the kinds" of tooth plugging employed; that would not only be laborious but uninteresting. But will give a few thoughts on the most approved method of operating.

Before proceeding further let me here remark, that to secure the best possible results, it is almost indispensably necessary to have the confidence of the patient, for without it, we had better not operate.

When a patient presents himself for an operation the first thing is to examine the condition of the teeth, mouth, and system. Ascertain whether he wants a part of his teeth, or all of them "fixed."

Some persons will apply to us to have one or two teeth filled, while others are needing our services, perhaps, more than those they wished filled. This, however, is not so often the case in city as in country practice. When it is the case that our patients wish only a partial operation, (as we will term it,) we should be the more careful in our examination. For it may be, to fill such teeth as they desire filled, and leave the other teeth unfilled, would do the patient little or no good, and the dentist real harm. In most cases, with this class of persons, they will want you to insure your work, *i. e.*, if the plug ultimately fails, they want another like it for nothing. You will find this kind of patient the hardest of all others to please. Should a plug fall out, they will tell it to every body they see, that Dr. A. plugged a tooth for them and it was worthless; thereby doing great injury to the operator. As these are considerations of much importance it is highly necessary not to leave anything unexamined. Never work for such persons unless you are sure the work will do more than you propose.

If your patients wish all done that will be of benefit to the teeth, then commence with the smallest and the easiest cavity of the molar teeth first. The object of this is, in beginning with the smaller cavities of the molar teeth, your operation will be less painful, so that should the patient be one of those nervous, sensitive persons often met with, by the time we reach the teeth most likely to give pain, their feelings have become so much enlisted, that they seldom complain; if they do, it is not so much as where you begin with the front teeth or those most sensative. And again, but few persons, after placing themselves in the hands of a dentist, will stop short of having their front teeth secured. Hence the importance of beginning the operation with the posterior teeth. The same will hold good in tooth extracting.

In filling teeth very much depends upon the preparation of the gold and the kind of instruments employed.

The best method of preparing gold foil for filling teeth is by folding the sheet twice, making four thicknesses, then for large cavities cut into two strips, and for smaller into three or four. These form into wisps or coils by corrugation, or gently working the edges in toward the center, being careful that it is not made too dense. These cut into blocks of various lengths, some as long as the diameter of the coil, others longer, or a little shorter. By this means you will have a variety of sizes, which will be very necessary in the progress of the operation. These should be kept in a box with a cover to it, so that when you are not using them, the dust may be prevented from lodging on them. These blocks are picked up on the point of the plugger, and carried through the flame of a spirit lamp, to drive out all the moisture, and then to the place intended for them. By thus preparing good gold foil, a plug may be made as solid as molten gold.

The points of the pluggers must be finely serrated for this kind of gold, and not too large. I prefer small pointed instruments, it takes a little longer to do the work, but it is much better when it is done.

In small or simple cavities, there is not usually much required, but to cleanse properly and fill, so far as manipulation is concerned after the cavity is prepared, I find very little difference. In simple cavities take the same care as in more difficult ones. Invest the tooth with a folded napkin as well as it can be, anneal the gold and pack it carefully as in any other shaped capity.

The second class of cavities demands more care, and will next claim our attention.

This class frequently consists of proximal cavities, and in a great many cases so crowded as to require separation before you have room to operate. The modes of separation usually resorted too are, the file, chisel and wedge, these should be adopted according to circumstances. In some cases the file is best, in others the chisel, and not unfrequently the wedge or rubber is preferable to either.

When the tooth is sufficiently removed to make room for the operation, form the margins of the cavity first, and then proceed to remove the decay. In some cavities of this class the decay leaves very shallow and sometimes scarcely no cavity at all. If the cavity be shallow with very poor walls, either cut grooves, or drill pits for retaining points. If pits are drilled for retaining points, fill each of them separately, and unite the plug to them. But should the decay be deep, the removal of which would expose the nerve, I would prefer leaving the decayed bone next to the nerve, unless it is soft and spongy, then would remove it. To leave the decayed bone of a tooth next to the nerve, if it be firm enough to resist the pressure of filling, will form a better protection to the nerve than any dentist can put there, it is better adapted, having no undue pressure upon the nerve, and if well saturated with creosote, will exert no deleterious effect upon the nerve or tooth.

We are not unfrequently called upon to fill a tooth that has lost half, and sometimes more of its crown, or perchance to extract it. When the latter is the case, we should be very careful, for should a patient apply to have a tooth extracted, and we induce him to have the tooth treated, he will expect us to do more than if he had called voluntarially to have the same thing done. Then he will say, "well I did not expect much more, but thought I would try it," seemingly indifferent about it. But should we recommend its retention and fail to obtain a perfect result, in most cases we are injured twice the amount of the fee obtained for it. I am always more careful in such cases, than in operations of less importance.

In the first place, if we recommend any particular operation, and fail to do what we fully expected in the beginning, and perhaps would have done, had the patient done his duty, or other things over which we had no control whatever, prevented it. Then the patient not considering those things, is as ready to complain as though we were responsible for the failure. And secondly, should the operation give any dissatisfaction, by a little soreness or otherwise, which is sometimes the case in very large plugs, the gold being a good conductor of heat will cause pain sometimes, on taking cold or hot substances into the mouth, or if there should be a disposition to periostitis, or from breaking a portion of the tooth, causing the plug to become loose or to fall out entirely, they never attribute it to anything but the unskillfullness of the operator, when indeed he is not at fault in any particular. You may insert a plug of the same size and shape, of an amalgam, and only charge them fifty cents or a dollar, and if it falls out before they get home, or causes the face to swell, or an abscess to form and every other evil attending such work, they will seldom say anything; but let it be a gold plug, one requiring skill and patience to produce, and if they lose it there is no epithet too vile to pronounce upon their dentist. The secret of all is, that a good gold filling will cost them from ten to thirty or forty dollars, while an amalgam filling inserted by those who do the most of that kind of work will only cost them from fifty cents to one and a half dollars. From these considerations aside from professional pride, we should never attempt an operation unless we have some assurance of success, or know who we are operating for, and that he appreciates our skill as a dentist, and then having the doubts all in our favor.

To secure success in all cases, it is necessary that every step taken should be thorough. The cavity must be well prepared from the bottom. The walls must to be solid, (especially for the molars where there is any pressure in mastication). The retaining points good, and above all the filling made perfect from the beginning. I had about as soon undertake to build up a crown of gold as to take a case where a portion of the crown is gone. In the gold crown we rely upon the perfectness of the operation, and strength of the gold, while in the other we are liable to place too much confidence in the support of the walls of the tooth, and often don't make as good work in consequence. My experience in such fillings is success. I never have to my knowledge lost a single plug of this kind while I have lost several of less importance. The reason I have for it is, in this class I do thorough work, in the other I am not so particular. To insure success dont be in too great a hurry. Take ample time, don't work when tired. If your job is not done, drop off awhile. It is better to spend an hour of each day for a week, than loose such a plug by being too hasty with the operation. Some will say "we can't afford so much time. It won't pay." To all such I would say, then don't work. Make your charges conform to the work, not the work to the charges. Consider the time and materials, and if your patrons are not willing to pay you a reasonable fee for them, then my advice would be either emigrate or quit the profession. In filling teeth of this character it is a very good plan to keep the centre or middle of the plug slightly fuller than next to the walls; you can more easily keep the filling close up to the walls. If, however, you follow the suggestions in the foregoing part of this paper, it is not so essential, for you will pack every part of the surface, as you proceed, and will be sure of making good work next to the walls. I am very particular about the plug fitting up firmly to the walls of the cavity, for a filling that does not is little better than none at all. In finishing a large plug, they, as well as smaller ones, should be built out a little fuller than the tooth, so as to have room for dressing down and finishing the surface.

When the nerve of a tooth is exposed and diseased, destroy it, if healthy treat it. When it is exposed and healthy, and the patient has good recuperative powers, treat with creosote and cap it. Sometimes if in excavating a cavity the nerve is exposed, I have simply dipped a piece of gold in creosote and laid it on the exposed point and fill over it. When I use the lead cap, I take a shot and beat it out to No. 20 or 22 American plate gauge, trim a piece large enough to cover the exposed nerve, and to lap upon the tooth about the thirty-second part of an inch.

To prepare the gold for a filling of this kind, roll the gold as in ordinary cases, then cut off four blocks to suit the size of the cavity, allowing the blocks to cover the cap at least half the distance between the cap and the walls of the tooth. If the cavity be small let the blocks extend out to the walls, and one block large enough to form a cap to the four blocks after they are placed in position. To secure the cap more readily use a little stiff mucilage on the bottom side, then place it on the nerve, and then one block anteriorly and one posteriorly, letting them lap at least one third of the size on the leaden cap. Then place the other two blocks at right angles with the first, allowing them to lap as before. Take the fifth block and lay it over the center, and condense the edges slightly. You now have an arch that with care in manipulation will resist sufficient force to make a solid plug. After the completion of the arch proceed as in ordinary cases.

There are other modifications and treatment that would be well to mention in a paper like this, did it not lengthen it too much. But I have detained this Association long enough already. Hoping this effort will be of some benefit to the younger portion, at least, I close.

